# Uremic Apelin and Leucocytic Angiotensin-Converting Enzyme 2 in CKD Patients

**DOI:** 10.3390/toxins12120742

**Published:** 2020-11-26

**Authors:** Bogusz Trojanowicz, Christof Ulrich, Matthias Girndt

**Affiliations:** Department of Internal Medicine II, Martin Luther University Halle-Wittenberg, 06120 Halle (Saale), Germany; christof.ulrich@uk-halle.de (C.U.); matthias.girndt@uk-halle.de (M.G.)

**Keywords:** apelin, ACE2, monocytes, uremia, atherosclerosis

## Abstract

Apelin peptides (APLN) serve as second substrates for angiotensin-converting enzyme 2 (ACE2) and, in contrast to angiotensin II (AngII), exert blood-pressure lowering and vasodilatation effects through binding to G-coupled APLN receptor (APLNR). ACE2-mediated cleavage of the APLN may reduce its vasodilatory effects, but decreased ACE2 may potentiate the hypotensive properties of APLN. The role of APLN in uremia is unclear. We investigated the correlations between serum-APLN, leucocytic APLNR, and ACE2 in 32 healthy controls (NP), 66 HD, and 24 CKD3–5 patients, and the impact of APLN peptides on monocytic behavior and ACE2 expression under uremic conditions in vitro. We observed that serum APLN and leucocytic APLNR or SLCO2B1 were significantly elevated in uremic patients and correlated with decreased ACE2 on uremic leucocytes. APLN-treated THP-1 monocytes revealed significantly increased APLNR and ACE2, and reduced TNFa, IL-6, and MCSF. Uremic toxins induced a dramatic increase of miR-421 followed by significant reduction of ACE2 transcripts, partially counteracted with APLN-13 and -36. APLN-36 triggered the most potent transmigration and reduction of endothelial adhesion. These results suggest that although APLN peptides may partly protect against the decay of monocytic ACE2 transcripts, uremic milieu is the most dominant modulator of local ACE2, and likely to contribute to the progression of atherosclerosis.

## 1. Introduction

Recently discovered apelin (APLN) peptides may act, in addition to angiotensin II, as substrates for angiotensin-converting enzyme (ACE) 2 [1,2,3]. The APLN gene encodes a precursor peptide of 77-amino acid [4], further cleaved to several active peptide-fragments, including APLN-36, -17, -13, and -12, which exert their actions via a G-protein coupled APLN receptor (APLNR) [5]. APLNR is widely expressed within the human body, including in the central nervous system, heart, liver, lung, kidney, and stomach. The presence of APLNR has also been confirmed in the vasculature, including endothelial and smooth muscle cells, and circulating leukocytes [6,7,8,9]. 

The APLN-APLNR-axis plays a pivotal role in cardiovascular regulation [10,11], thrombotic diseases [12], and processes of angiogenesis [13], and it seems that APLN exerts cardioprotective effects, at least in the animal models [14,15,16]. 

On the other hand, the APLN-APLNR system has been linked with inflammation and oxidative stress. Hashimoto et al. demonstrated that APLN is able to increase the proliferation of vascular smooth muscle cells, and APLN deficiency acts preventively against oxidative stress-linked atherosclerosis [17]. In the studies by Daviaud et al., TNFa, one of the main pro-inflammatory cytokines, was able to upregulate APLN expression in human and mouse adipose tissue [18]. In addition, exposure of hepatic stellate cells and hepatocytes to LPS or TNFa led to increased levels of APLNR [19]. Human umbilical vein endothelial cells reacted to APLN treatment with increased expression of intercellular adhesion molecule-1 (ICAM-1), vascular cell adhesion molecule-1 (VCAM-1), and monocyte chemoattractant protein-1 (MCP-1) [20].

Systemic chronic inflammation, progressive atherosclerosis, and related cardiovascular diseases are frequently observed in patients with chronic kidney disease (CKD) [21,22]. Diminished levels of ACE2 and the upregulation of ACE found on leukocytes of CKD patients, especially monocytes, may relevantly contribute to the progression of atherosclerosis. It has been demonstrated that primary or THP-1 monocytes respond to uremic conditions with decreased ACE2 levels, increased expression of ACE, and augmented endothelial-adhesion and transmigration [23,24,25]. 

Based on our previous data that ACE2 is diminished on CKD leukocytes and monocytes, and that the APLN/APNR-axis may be related to atherosclerosis and inflammation, we aimed to investigate the correlation between serum-APLN, leucocytic APLNR, and ACE2 expression in CKD patients. As the second objective of this study, we investigated the leucocytic expression of leucocytic organic anion transporter SLCO2B1 as a possible mediator of intracellular influx of uremic toxins, which in turn may regulate particular micro RNAs, especially miR-421. Furthermore, we tested the impact of uremic toxins on miR-421-mediated transcriptional decay of ACE2 in THP-1 monocytes. Finally, we investigated the effects of APLN peptides on monocytic expression of ACE2 and behavior of the monocytes under uremic conditions in vitro. 

## 2. Results

### 2.1. Levels of Serum APLN Are Elevated in CKD3–5 and HD Patients and Correlate with Leucocytic Expression of APLNR and ACE2

**Figure 1:**
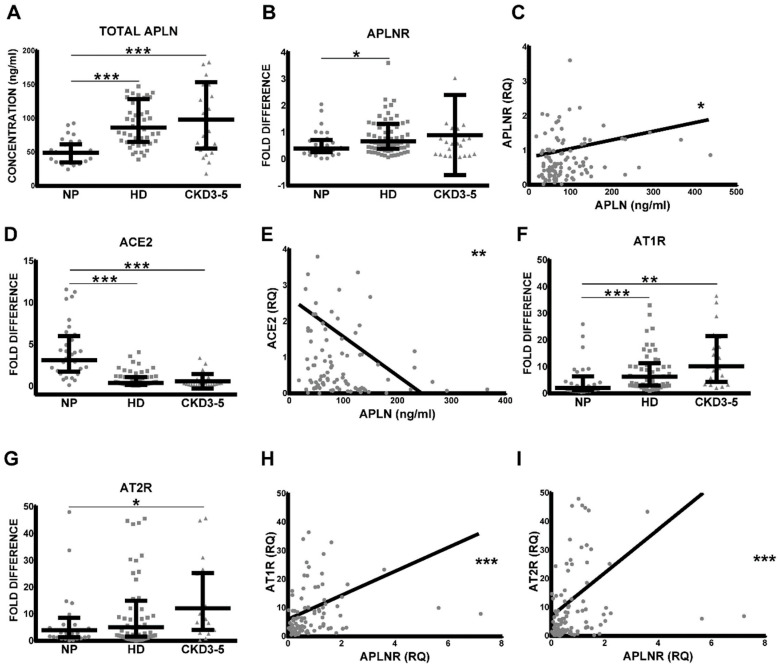
(**A**) Concentration of serum levels of total apelin (APLN) and leucocytic expression of (**B**) APLN receptor (APLNR), (**D**) angiotensin-converting enzyme 2 (ACE2), (**F**) angiotensin II receptor type 1 (AT1R), and (**G**) AT2R in healthy controls (NP), HD and non-dialyzed CKD3–5 patients. The levels of circulating serum APLN were correlated with leucocytic (**C**) APLNR and (**E**) ACE2. Correlations of leucocytic APLNR were performed with leucocytic (**H**) AT1R and type II (**I**) AT2R; fold difference values were calculated by comparison to expression of the target transcript in the reference sample and for the evaluation purposes was set as 1; medians with IRQs; *p*-values * <0.01, ** <0.001, and *** <0.0001 indicate statistical significance (Bonferroni correction was applied). Administration of AngII receptor blocker to our CKD patients resulted not only in some degree of reduction of leucocytic AT1R and AT2R, as demonstrated previously [22], but also serum APLN and leucocytic APLNR (Figure S2A,C).

As demonstrated in Figure 1A, the level of total serum APLN was significantly elevated in CKD3–5 and HD patients as compared with corresponding NP. Similar differences could be shown for APLNR expression patterns on circulating leucocytes; however, differences between NP and CKD3–5 were not significant. As demonstrated in Figure 1B, the leukocytes obtained from CKD3–5 and HD patients revealed noticeably elevated expression of APLNR as compared with NP. Furthermore, the levels of serum APLN correlated positively and significantly with the expression of leucocytic APLNR, ACE2 (Figure 1C–E), AT1R, AT2R (Figure S1D), and MASR (Figure S1C,E). Leucocytic APLNR correlated with leucocytic AngII and Ang1–7 receptors (Figure 1F–I and Figure S1A,B). 

### 2.2. Leucocytic Organic Anion Transporter SLCO2B1 Is Increased in Uremic Patients and Correlates with Diminished ACE2 

**Figure 2:**
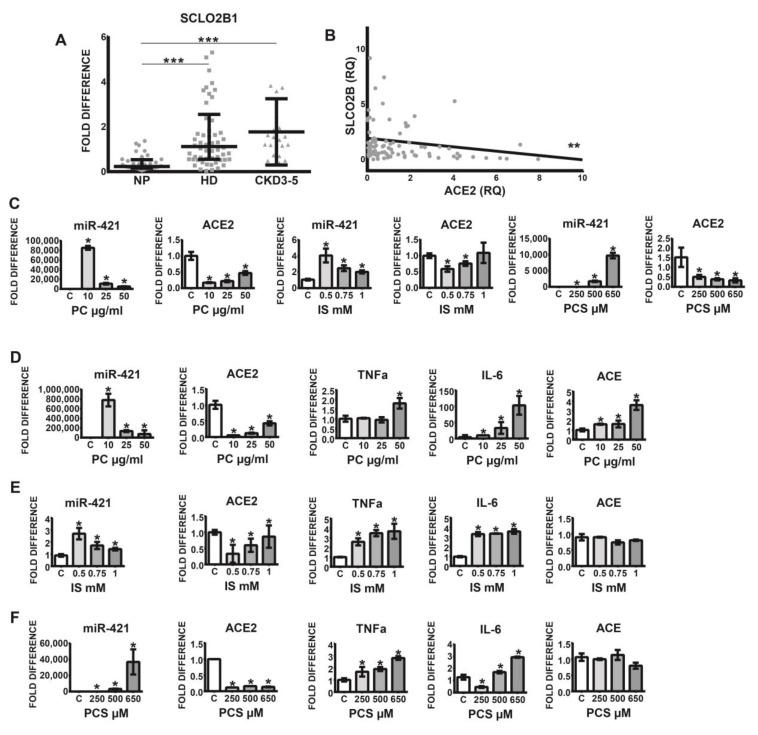
(**A**) Expression of organic-anion transporter SCLO2B1 in leucocytes obtained from healthy controls (NP), HD, and non-dialyzed CKD3–5 patients. (**B**) Correlation between leucocytic expression of ACE2 and SCLO2B1; fold difference values were calculated by comparison to expression of the target transcript in the reference sample and for the evaluation purposes was set as 1; medians with IRQs; *p*-values * <0.01, ** <0.001, and *** <0.0001 indicate statistical significance. (**C**–**F**) Regulation of monocytic miR-421, ACE2, TNFa, IL-6, and ACE treated with uremic toxins. (**C**) THP-1 monocytes were treated with *p*-Cresol (PC), indoxyl sulfate (IS), and *p*-Cresyl sulfate (PCS) in medium supplemented with 35 g/mL BSA and investigated for miR-421 and ACE2 expression. (**D**–**F**) THP-1 monocytes were treated with PC, IS, and PCS in medium without BSA and investigated for the expression of corresponding transcripts; means ± SD of five independent experiments; fold difference values were calculated by comparison to expression of the target transcript in the control sample and for the evaluation purposes was set as 1; * *p* < 0.05 as compared to C (control).

Previous findings indicate that leucocyte-derived macrophages, in addition to endothelial cells, smooth muscle cells, and osteoblasts, express several organic anion transporters, especially SLCO2B1, capable of uptake of indoxyl sulfate. Since this transporter plays a pivotal role in the induction of oxidative stress and the stimulation of pro-inflammatory cytokines, we investigated SLCO2B1 expression on the leucocytes originating from healthy volunteers and uremic patients. As demonstrated in Figure 2A, leucocytic SLCO2B1 is significantly elevated in CKD3–5 and HD patients as compared with corresponding NP individuals. Furthermore, increased expression of this organic transporter correlated significantly with decreased levels of leucocytic ACE2 (Figure 2B). 

### 2.3. Uremic Toxins Mediated Increase of Intracellular miR-421 Leads to Monocytic Silencing of ACE2 and Elevation of Pro-Inflammatory TNFa and IL-6

Since cells from uremic patients express high levels of leucocytic organic anion transporter SLCO2B1, the influx of uremic toxins into the cells may be elevated, leading to various pathophysiological events. Figure 2C–F demonstrates that treatment of THP-1 monocytes with uremic toxins, indoxyl sulfate (IS), *p*-Cresol (PC) led to noticeable upregulation of intracellular miR-421 followed by a significant decrease of ACE2 transcripts. This was associated with increased levels of pro-inflammatory TNFa and IL-6 (Figure 2D–F). Note that ACE transcripts were upregulated under PC treatment only (Figure 2D).

### 2.4. Uremic Milieu and APLN Peptides Increase the Expression of APLNR In Vitro

**Figure 3:**
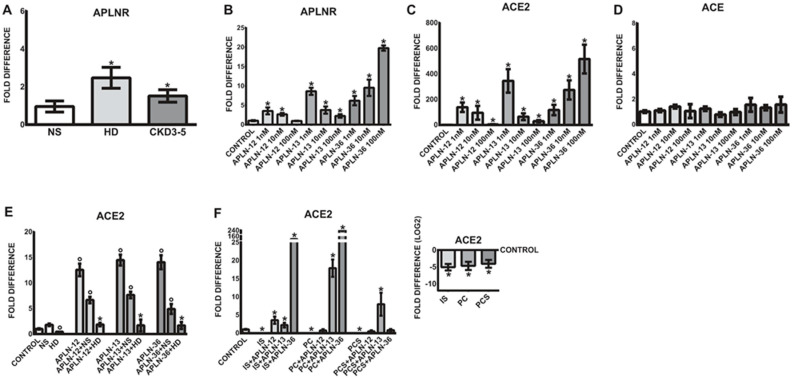
Expression of APLNR and ACE2 in THP-1 monocytes treated with patients’ serum, APLN peptides, or uremic toxins. (**A**) THP-1 cells were incubated with 10% of pooled sera obtained from healthy volunteers (NS), HD, and non-dialyzed CKD3–5 patients and investigated for the expression of APLNR. (**B**–**D**) Monocytic expression of APLNR, ACE2, or ACE under treatment with different concentrations of APLN-12, -13, and -36. (**E**) Expression of monocytic ACE2 in THP-1 cells treated with pooled NS or HD sera or co-treated with APLN peptides; means ± SD of five independent experiments; * *p* < 0.05 as compared to HD; *p* < 0.05 as compared to control or NS. (**F**) Expression of ACE2 in the cells treated with uremic toxins alone or co-treated with APLN peptides; note that ACE2 expression in uremic toxins treatment is also presented separately (values are log_2_ transformed, control set as 0); means ± SD of five independent experiments; fold difference values were calculated by comparison to expression of the target transcript in the control sample and for the evaluation purposes were set as 1; * *p* < 0.05 as compared to NS or control, respectively.

As demonstrated in Figure 3A, uremic sera led to significantly upregulated expression of APLNR as compared to treatments with NS serum. 

To test whether APLN peptides are able to induce similar expression pattern as observed in vivo, THP-1 monocytes were treated with different concentrations of APLN-36, -13, and -12. As demonstrated in Figure 3B, APLN peptides significantly upregulated the expression of APLNR in human monocytes in a concentration-dependent fashion as compared to untreated controls. The most potent concentration for APLN-12 and -13 was 1 nM, while the most pronounced upregulation of APLNR was observed with APLN-36 at 100 nM.

### 2.5. APLN Peptides Dramatically Increase the Expression of ACE2 and APLNR

As shown in Figure 3C, similar to APLNR regulation, APLN peptides significantly elevated ACE2 expression in THP-1 monocytes as compared to corresponding controls. The most effective concentration for APLN-12 and -13 was 1 nM, while the strongest upregulation of ACE2 was observed with APLN-36 at 100 nM. It is worth noting that monocytic ACE expression remained unaffected under all APLN treatments (Figure 3D).

### 2.6. Uraemia-Mediated Decrease in Monocytic ACE2 Could Be Partially Re-Induced with APLN Peptides 

Based on the previous observations that uremic serum significantly reduces the expression of ACE2 in THP-1 and human primary monocytes, we tested the impact of uremic milieu and selected uremic toxins on APLN-triggered expression of ACE2. As demonstrated in Figure 3C, THP-1 monocytes reacted to all APLN treatment with significantly increased expression of ACE2. Treatment of these cells with uremic serum or the uremic toxins IS, PC, or PCS led to significantly diminished expression of ACE2 (Figure 3E,F). Co-treatment of cells under uremic conditions with APLN peptides partially re-induced the expression of ACE2. The presence of all APLN peptides in uremic serum was able to elevate the ACE2 expression to a level similar as in the treatments with normal serum only (Figure 3E). The results suggest that all APLN peptides may induce ACE2 expression, but the presence of uremic conditions in the form of patient’s serum dramatically decreases their effects.

In the presence of uremic toxins, IS, PC, and PCS, THP-1 monocytes also reacted with noticeably decreased expression of ACE2 (Figure 3F). Co-treatment of these cells with particular APLN peptides was able to re-induce the ACE2 expression more effectively than in the presence of patient’s serum, especially in THP-1 monocytes incubated with APLN-36 in the presence of IS and PC, and APLN-13 in the presence of PCS. 

### 2.7. APLN Peptides Decrease the Expression of TNFa and IL-6 In Vitro as well as AngII and Ang1–7 Receptors 

**Figure 4:**
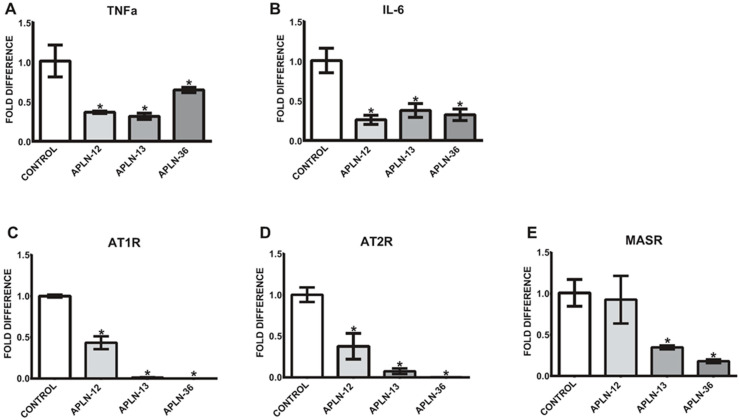
Expression of TNFa (**A**), IL-6 (**B**), AT1R (**C**), AT2R (**D**), and MASR (**E**) in THP-1 monocytes treated with APLN-12, -13, and -36 peptides; means ± SD of five independent experiments; * *p* < 0.05 as compared to control.

Since the levels of total APLN peptides are significantly elevated in uremic serum, we aimed to test whether treatment of the cells with APLN-12, -13, and -36 would be able to affect the expression of TNFa and/or IL-6. As demonstrated in Figure 4A,B, the levels of TNFa and IL-6 were noticeably decreased in THP-1 monocytes under 72-h treatments, independently of the APLN peptides concentrations used. Further, treatment with APLN peptides led to a significant decrease in the expression of AngII and Ang1–7 receptors, as demonstrated in Figure 4C–E.

### 2.8. APLN Peptides Affect Transmigration and Adhesion of the Cells In Vitro

**Figure 5:**
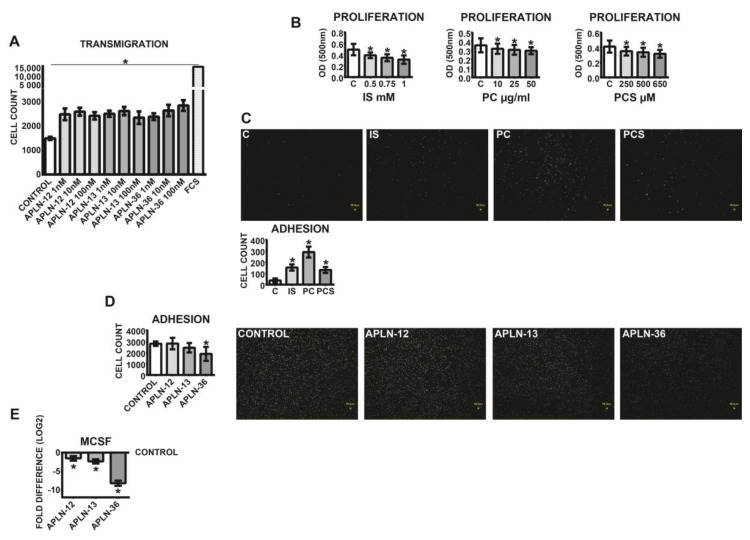
Transmigration, proliferation, and endothelial adhesion of THP-1 monocytes incubated with APLN-12, -13, and -36 peptides or uremic toxins. (**A**) APLN-triggered transmigration of the monocytes through 3 µM pore size inserts. The cells were transmigrated for 24 h and counted with a flow cytometer; transmigration under FCS served as a positive control; means ± SD of five independent experiments; * *p* < 0.05 as compared to control. (**B**) Proliferation of THP-1 monocytes under treatment with *p*-Cresol (PC), indoxyl sulfate (IS), and *p*-Cresyl sulfate (PCS). (**C**,**D**) Adhesion of the monocytes to endothelial monolayers under (**C**) uremic toxins or (**D**) APLN peptides. Calcein-labeled cells were pre-treated with APLN peptides or uremic toxins and incubated for 30 min in the presence of endothelial monolayers at the chamber bottom. Adhered cells were visualized with fluorescent microscopy and counted; means ± SD of cell number in 10 microscopic fields in three independent experiments. Representative images are shown. (**E**) Expression of MCSF in the monocytes treated with APLN peptides; values are log_2_ transformed, control set as 0; means ± SD of five independent experiments; fold difference values were calculated by comparison to expression of the target transcript in the control sample and for the evaluation purposes were set as 1; * *p* < 0.05 as compared to control. Proliferation was performed with MTT and measured at 500 nm; means ± SD of five independent experiments; * *p* < 0.05 as compared C (control).

In order to investigate the functional effects of APLN peptides, THP-1 monocytes were subjected to transmigration and adhesion assays. As demonstrated in Figure 5A, all APLN treatments led to significantly elevated transmigration rates of THP-1 monocytes as compared to the control. The most potent inductor of transmigration was APLN-36 at a concentration of 100 nM. 

Investigations of vitality performed with MTT assay, which depends on the cellular metabolic activity, revealed that the medium milieu under all uremic toxins tested led to decreased proliferation rates of the cells (Figure 5B). Further investigations demonstrated that human THP-1 monocytes pre-treated with uremic toxins reacted with noticeably elevated adhesion to endothelial HUVEC monolayers (Figure 5C). The opposite effects could be induced with APLN peptides; in particular, treatment of the cells with APLN-36 led to noticeably diminished adhesion of the monocytes (Figure 5D) and downregulation of MCSF (Figure 5E).

## 3. Discussion

This study demonstrates that CKD patients possess significantly elevated levels of serum APLN which correlate significantly with increased expression of its receptor, APLNR, and decreased ACE2 in circulating leukocytes. Since the local RAS is obviously overactivated in patients with renal failure, it seems that the induction of the APLN/APLNR axis may influence the AngII-mediated pathophysiological effects. Chun et al. showed that AngII-triggered proatherosclerotic effects may be abrogated with APLN infusion in the ApoE-KO mouse model. The authors postulated that such an inhibitory modulation of AngII signaling may occur through physical interaction between APLNR and AT1R, independent of APLN [26]. In accordance with these observations, we were able to demonstrate that increased leucocytic expression of APLNR in CKD patients correlated significantly not only with elevated levels of both AngII receptors, but also with increased expression of MASR, the receptor for Ang1–7. Furthermore, administration of AngII receptor blockers to our CKD patients resulted not only in some degree of reduction of leucocytic AT1R and AT2R, but also APLNR and serum APLN [22]. On the other hand, administration of ACE inhibitors was related with increased levels of circulating Ang1–7 and, presumably, ACE2 [27].

In further studies on APLN-KO mice, pharmacological or genetic inhibition of AT1R rescued not only impaired heart contractility and hypertrophy, but also restored the ACE2 levels. The same effects could be achieved with Ang1–7 or APLN treatment, which resulted in increased ACE2 expression and promoter activity, as seen in AT1R-deficient mice [28]. Our data in vitro demonstrated that treatment of THP-1 monocytes with APLN peptides led not only to upregulation of ACE2, but also to elevated levels of APLNR. It is worth noting that APLN-mediated induction of ACE2 expression was not related with ACE regulation. On the other hand, although uremic patients possess significantly higher serum levels of APLN than healthy individuals, leucocytic expression of ACE2 in these patients significantly decreased, while ACE as well as AngII and MAS receptors remained upregulated. Since leucocytic expression of these receptors does not correlate with circulating levels of APLN and ACE2 expression is diminished in CKD patients, it seems that uremic modulation of APLN/APLNR axis is more complex. 

We demonstrated that THP-1 cells pre-treated with uremic serum that contains more APLN than healthy ones reacted with increased expression of APLNR and MASR, and decreased levels of ACE2 [23]. Treatment of the same cells with APLN peptides also led to elevated levels of APLNR, but dramatically upregulated expression of ACE2 and decreased levels of MASR. Given that higher contents of AngII or APLN in uremic conditions regulate the expression of corresponding receptors, but are not able to induce the leucocytic ACE2 or serum Ang1–7 [27], it seems that other hindering factors are involved. This fact is further supported by our in vitro data demonstrating that in the presence of uremic serum, an APLN-mediated increase in monocytic ACE2 expression is dramatically reduced. However, a uremic toxin-mediated decrease in monocytic ACE2 may be partially re-induced with APLN peptides, more effectively than in the presence of patients’ serum. 

Wang et al. identified neutral endopeptidase (NEP), which is upregulated in human abdominal aortic aneurysm tissue as a major enzyme that metabolizes and inactivates APLN peptides. Furthermore, APLN suppression in this model was related to decreased levels of ACE2 [29]. Since NEP is the key enzyme hindering APLN-mediated actions, we speculate that the presence and/or over-activation of NEP in uremic conditions may, in addition to the classical toxins, diminish the beneficial effects of ACE2. It is worth noting that inhibition of NEP which degrades the peptides of natriuretic system may have beneficial effects in patients with CKD [30]. On the other hand, studies by Gutta et al. revealed that in diabetic CKD patients with macro-albuminuria, plasma levels of NEP are significantly decreased as compared to healthy subjects or diabetic patients with normo- or micro-albuminuria [31]. Since the levels of NEP as APLN-inactivating enzymes are decreased in uremic patients, the higher levels of APLN in HD and CKD3–5 groups followed by an ACE2 increase could be expected. On the other hand, as demonstrated by our data, uremic serum or addition of APLN to this serum were not able to induce ACE2. We speculate that higher levels of AngII in CKD patients serve in the first line as substrate for ACE2 and override APLN-directed activity. Furthermore, ACE2 activity is dramatically decreased in uremic serum as demonstrated by diminished Ang1–7 levels. We also cannot also exclude the possibility that uremic conditions may in some way modify APLN peptides masking their availability for target enzymes.

The impact of uremic toxins on different RAS components was reported previously, including behavior of renal or endothelial cells [32,33,34,35], but their role in APLN-mediated regulation of ACE2 in leucocytes, especially monocytes, is unclear. Our results revealed that all uremic toxins tested increased not only the endothelial adhesion of the monocytes, but also led to diminished proliferation of these cells.

Nakano et al. demonstrated that human macrophages possess a fully functional system of various organic anion transporters. The authors revealed that IS uptake and cellular activation of human macrophages, resulting in increased expression of pro-inflammatory cytokines, was mainly mediated by SLCO2B1 transporter and participation of Dll4-Notch signaling. Furthermore, targeting this pathway with siRNA encapsulated in macrophage-targeted lipid nanoparticles or by Dll4 neutralizing antibody resulted in diminished atherosclerotic events in CKD mice [36]. In accordance with these results, our data revealed that leucocytes obtained from HD and especially non-dialyzed CKD patients possess noticeably elevated expression of SLCO2B1 transcripts as compared to the healthy population. We suggest that the augmented presence of the OATP2B1 transporter, encoded by SLCO2B1, on the leucocytes, especially monocytes, may lead to the increased influx of the uremic toxins into the cells and support development of various pathophysiological events. In accordance with these findings, we demonstrated that particular uremic toxins are able to induce the intracellular monocytic levels of miR-421 which in turn targets and decreases the transcripts of ACE2 [27]. This reduction can be partially counteracted with APLN-mediated induction of ACE2, even in the presence of uremic serum. 

Experimental treatment of the monocytes with APLN peptides, especially APLN-36 led not only to the diminished pro-atherosclerotic events in the form of reduction of endothelial-adhesion and increased transmigration, but also decreased levels of MCSF and pro-inflammatory TNFa and IL-6. It is worth noting that in a chronic mouse model of obesity and impaired glucose tolerance, APLN-36 reduced body weight, blood glucose levels, and serum cholesterol more effectively than APLN-13. In comparison to the wild type of APLN-36, the variants of APLN-36 with a single amino acid substitution (L28A) or longer (L28C-30 kDa-PEG) preserved most of their metabolic properties, but were not able to lower blood pressure [37]. With regard to renal insufficiency, whether both cardiovascular and/or metabolic biological activities of APLN-36 are required to slow cardio-renal disease progression in CKD patients is currently unclear and should be addressed in the future. Furthermore, the effects of the potential inhibitor of several solute carriers, for example probenecid, on monocytic SLCO2B1 and pro-atherosclerotic events are not clear and were not tested here, but may offer an interesting therapy in decreasing the uremic effects in CKD patients.

In conclusion, although APLN peptides may act against uremic-driven disruption of monocytic ACE2, uremic milieu overrides this protective mechanism, thus contributing to the progression of atherosclerosis.

## 4. Materials and Methods

### 4.1. Patients, RNA, and Serum Isolation 

Total blood RNA and serum were isolated from chronic HD patients treated at the Dialysis Ward of the University Hospital Halle, CKD3–5 patients not on dialysis, and healthy volunteers (NP). All HD patients were free of acute infectious complications, not on immunosuppressive medication, and were treated three times weekly according to HD conditions mentioned previously [22]. All HD blood samples were obtained prior to a regular HD session from the dialysis access. 

CKD3–5 non-dialyzed patients and NP were classified according to their eGFR (according to CKD-EPI formula). CKD3-5 patients were treated at the Department of Internal Medicine of the University Hospital Halle. 

HD, CKD3–5 patient’s, and NP characteristics are presented in Table 1.

The study was approved by the ethical committee of the Martin Luther University, Faculty of Medicine under the number 2014-20 on 7 October 2014. All patients and persons involved in this study gave written consent. The study was performed in accordance with the rules of the Declaration of Helsinki.

### 4.2. Serum APLN Levels

Total levels of circulating serum APLN were measured with the APLN C-Terminus Enzyme Immunoassay (EIA) Kit (RAB0018, Sigma Aldrich, Munich, Germany) in accordance with the manufacturer’s instructions. 

### 4.3. Cell Culture and Treatments of THP-1 Monocytes

In this experiment, 1 × 10^6^/well THP-1 monocytes were treated with RPMI (Sigma Aldrich) medium containing 10% of pooled HD or CKD3–5 or NP sera for 72 h. These three separate serum-pools were created by mixing of 50 μL of all HD or all CKD3–5 or all NP sera originating from individuals mentioned in Table 1. Additionally, THP-1 monocytes were treated and/or co-treated with 0.5, 0.75, or 1 mM indoxyl sulfate (IS; Sigma Aldrich), 10, 25, or 50 µg/mL *p*-Cresol (PC; Sigma Aldrich) and 250, 500, or 650 µM *p*-Cresyl sulfate (PCS; Tokyo Chemical Industry, Tokyo, Japan) for 24 h in serum-free RPMI medium, in accordance with the experimental set up. Initial treatments of THP-1 monocytes and resulting miR-421 and ACE2 expression were performed on the cells incubated in the medium supplemented with 35 g/L BSA (Sigma Aldrich). Since there was not significant differences in the expression analyses as compared to the treatments without albumin, BSA supplementation was omitted in further investigations.

APLN treatments were performed with 1, 10, and 100 nM APLN-36 (Phoenix Europe, Karlsruhe, Germany), -13 (Bachem, Bubendorf, Switzerland), and -12 (Bachem) for 24 h in RPMI medium without FCS. Co-treatments of serum- or uremic-toxin-treated monocytes were performed with 1 nM APLN-12 or -13, or 100-nM APLN-36. 

Total RNA was isolated by employment of the Direct-zol RNA Kit (Zymo Research Europe, Freiburg, Germany) in accordance with the manufacturer’s instructions. 

### 4.4. Real Time PCR 

Amplifications of TaqMan-based (Thermo Fisher Scientific, Schwerte, Germany) APLNR (Hs00270873_s1), SLC02B1 (Hs01030343_m1), ACE (Hs00174179_m1), MASR (Hs00267157_s1), TNFa (Hs01113624_g1), IL-6 (Hs00985639_m1), ACTB (Hs99999903_m1), and RPL37A (Hs01102345_m1) were performed with qPCRBIO Probe Mix Hi-ROX (Nippon Genetics, Dueren, Germany) in a StepOne plus System (Thermo Fisher Scientific). ACTB and RPL37A (Ribosomal Protein 37a) were employed for normalization. Amplifications of ACE2, AT1R (angiotensin II type I receptor), AT2R (angiotensin II type II receptor), and RPL37A were performed with 5x HOT FIREPol^®^ EvaGreen^®^ qPCR Mix Plus (Solis BioDyne, Tartu, Estonia) and sequences in accordance with [23]. Primer sequences for ACE2 were: S-5′CAT TGG AGC AAG TGT TGG ATC TT, AS-5′GAG CTA ATG CAT GCC ATT CTC A. Data evaluation was performed with DataAssist Software (Thermo Fisher Scientific). 

Total RNA for intracellular, monocytic miR-421 was reversely transcribed with TaqMan Advanced miRNA cDNA Synthesis Kit (Thermo Fisher Scientific) and investigated with RT-PCR by employment of TaqMan (Thermo Fisher Scientific) probes specific for miR-421 (478088_mir) and miR-361-5p (478056_mir), used to normalize the levels of miR-421.

### 4.5. Transmigration Assay

Next, 5 × 10^5^ THP-1 cells were re-suspended in 400 µL RPMI medium and seeded into upper transmigration chamber (Millicell Cell Culture Inserts (Millipore, Darmstadt, Germany, 3 µM pore size, 12 mm diameter). The cells transmigrated 24 h through membranes towards the lower chamber filled with 600 µL RPMI medium supplemented with 1, 10, and 100 nM APLN-36, -13, and -12, 10% FCS as control. Transmigrated cells from the lower chamber were counted by flow cytometry (MACSQuant, Miltenyi, Bergisch Gladbach, Germany). The experiment was repeated at least three times.

### 4.6. Adhesion Assay

Then, 1 × 10^6^ THP-1 cells were re-suspended in control RPMI or RPMI medium supplemented with 1 nM of APLN-12 and -13 and 100 nM of APLN-36 or RPMI medium supplemented with 0.5 mM (~125 µg/mL) IS, 50 µg/mL PC, and 650 µM (~122 µg/mL) PCS, and subsequently incubated for 72 h. Note that control cells for the uremic toxins experiment received corresponding amounts of methanol. Thereafter, THP-1 cells were labeled with 1x Calcein-AM (Cayman Chemicals, Ann Arbor, MI, USA) and added to HUVEC (Lonza, Basel, Switzerland) monolayers (3 × 10^5^ cells/well). The plates were incubated for 30 min at 37 °C, and thereafter the monolayer was gently washed three times with RPMI. Adherent monocytes were photographed using a Biozero BZ-9000 fluorescence microscope (Keyence, Neu-Isenburg, Germany). The number of the adherent cells was evaluated in 10 microscopic fields for each situation by employment of Image-Pro plus software (Media Cybernetics, Rockville, MD, USA). All experiments were repeated at least three times. 

### 4.7. Statistics

Each experiment was repeated at least three times. Data are presented as mean ± SD or median with interquartile range. Distribution of the quantitative variables was tested using D’Agostino-Pearson omnibus, Shapiro–Wilk, or Kolmogorov–Smirnov normality tests. If indicated, log transformations were applied. Depending on data distribution, parametric (differences between paired values are consistent) or non-parametric (Wilcoxon matched-pairs signed rank test) two-sided *t*-tests were used. For multiple comparisons within parametric and non-parametric tests, a corresponding Bonferroni correction was applied. Additionally, group analyses by employment of one-way ANOVA were performed. Values of *p* < 0.05, <0.01, <0.001, and <0.0001, depending on the experiment, were considered to represent statistically significant differences. GraphPad Prism and SPSS software were used for statistical analyses.

### 4.8. Limitations

Although this study was performed on the samples originating from healthy controls and uremic patients, we must indicate several limitations.

By design, the expression of the leucocytic parameters was tested on the transcript level only. Due to the protein sequence of APLN and technical nature of ELISA, we were not able investigate the specific APLN peptides in patients’ serum. This issue should be addressed in the future clinical trials. 

## Figures and Tables

**Table 1:** Biometric characteristics of patients in control (NP), HD, and CKD3–5 groups.

Parameters	NP (*n* = 32)	HD (*n* = 66)	CKD3–5 (*n* = 24)
Age (years)	53.1 ± 6.84	63.02 ± 14.69	74.54 ± 10.72
Male/female	8/24	44/22	8/16
Body mass index (kg/m^2^)	24.50 ± 4.01	25.91 ± 4.73	28.56 ± 6.42
Hypertension (%)	6.25	92.42	91.67
Diabetes (%)	3.12	37.88	33.3
Ever smoker (%)	46.88	57.58	41.67
Dialysis vintage HD (years)	0	8.40 ± 5.11	0
History of cardiovascular disease (%)	0	45.45	66.67
Creatinine (µmol/L)	69.88 ± 12.94	763.58 ± 290.65	157.71 ± 67.28
Urea (mmol/L)	4.54 ± 1.36	21.57 ± 6.95	15.33 ± 17.48
Albumin (g/dL)	4.10 ± 0.30	3.88 ± 0.48	N/A
C-reactive protein (mg/L)	1.73 ± 1.36	13.38 ± 23.84	11.03 ± 15.13
Leucocytes (G/L)	6.15 ± 1.59	7.69 ± 3.44	6.71 ± 1.75
Monocytes (% leukocytes)	8.22 ± 2.41	8.48 ± 2.99	N/A
HDL (mmol/L)	1.78 ± 0.45	1.32 ± 0.63	N/A
LDL (mmol/L)	2.42 ± 0.92	3.78 ± 0.86	N/A
Cholesterol (mmol/L)	6.17 ± 0.83	4.37 ± 1.26	N/A
Triglyceride (mmol/L)	1.40 ± 1.14	2.20 ± 2.36	N/A
25(OH) vit. D (%)	0	90.91	25.00
ACEi (%)	0	24.24	33.33
ARB (%)	0	42.42	45.83
β-blockers (%)	0	77.27	75.00
Statins (%)	0	37.88	58.33

N/A, not available.

## Supplementary Materials

The following are available online at https://www.mdpi.com/2072-6651/12/12/742/s1, Figure S1: Expression of (A) leucocytic MAS receptor (MASR) and its correlation with (B) leucocytic APLNR. Correlation between serum APLN and (C) AT1R, (D) AT2R, and (E) MASR; fold difference values were calculated by comparison to expression of the target transcript in the reference sample and for the evaluation purposes were set as 1; medians with IRQs; *p*-values * <0.01, ** <0.001, and *** <0.0001 indicate statistical significance (Bonferroni correction was applied). Figure S2: Serum (A) APLN and (B) Ang1–7 and (C) leucocytic expression of APLNR in CKD patients treated with AngII receptor blocker (ARB+) or angiotensin-converting enzyme inhibitor (ACEi+); levels of serum Ang1–7 were measured as mentioned previously [27]. (D) Serum APLN in healthy controls (NP), HD, and non-dialyzed CKD3–5 patients; fold difference values were calculated by comparison to expression of the target transcript in the reference sample and for the evaluation purposes were set as 1; medians with IRQs; *p*-values * <0.01, ** <0.001, and *** <0.0001 indicate statistical significance (Bonferroni correction was applied).
