# Supplementary material for: Uremic Apelin and Leucocytic Angiotensin-Converting Enzyme 2 in CKD Patients
Source: Toxins (Basel). 2020 Nov 26;12(12):742. doi: 10.3390/toxins12120742 (PMC7760850; doi:10.3390/toxins12120742)
Supplement: Supplementary file 1 [file toxins-12-00742-s001.pdf]

Supplementary Materials: Uremic Apelin and Leucocytic Angiotensin-Converting Enzyme 2 in CKD Patients

Bogusz Trojanowicz, Christof Ulrich and Matthias Girndt

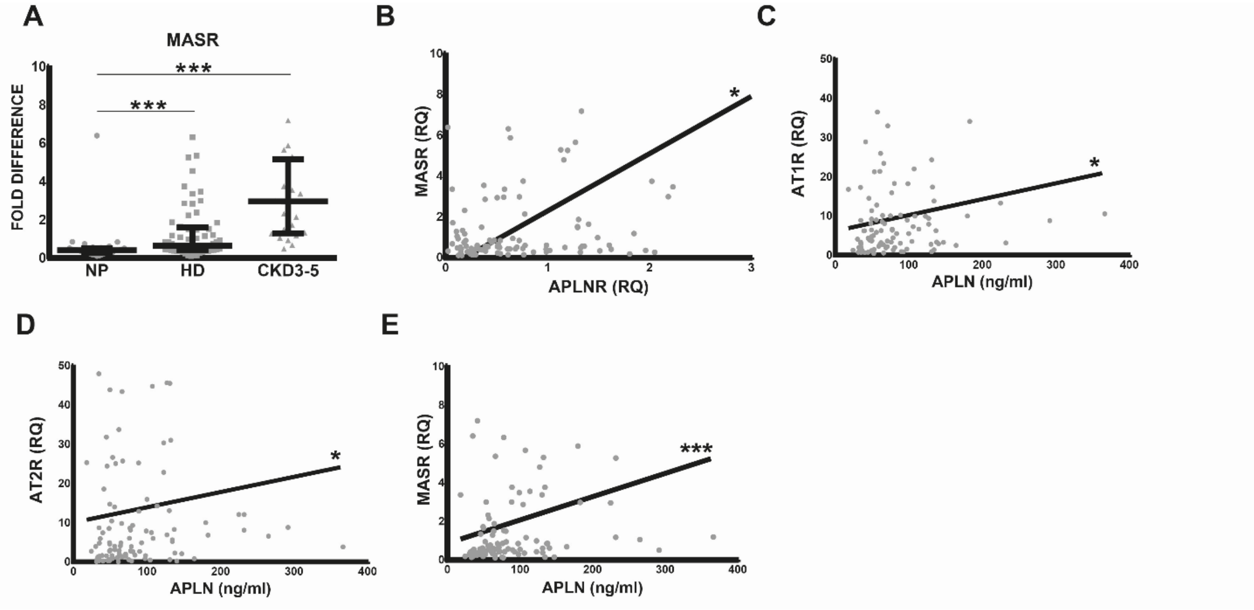

Figure S1. Expression of (**A**) leucocytic MAS receptor (MASR) and its correlation with (**B**) leucocytic APLNR. Correlation between serum APLN and (**C**) AT1R, (**D**) AT2R and (**E**) MASR; fold difference values were calculated by comparison to expression of the target transcript in the reference sample and for the evaluation purposes was set as 1; medians with IRQs; p values * < 0.01, **<0.001 and ***<0.0001 indicate statistical significance (Bonferroni correction was applied).

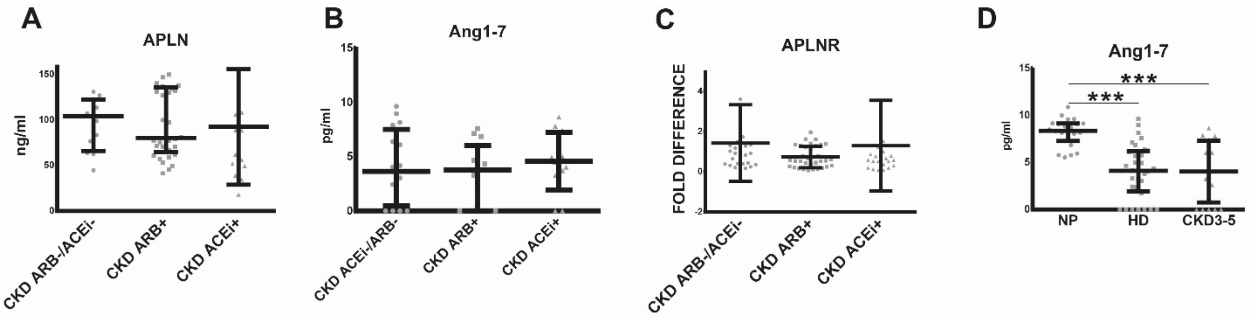

Figure S2. Serum (**A**) APLN and (**B**) Ang1-7 and (**C**) leucocytic expression of APLNR in CKD patients treated with AngII receptor blocker (ARB+) or Angiotensin Converting Enzyme Inhibitor (ACEi+); levels of serum Ang1-7 were measured as mentioned previously [27]. (**D**) Serum APLN in healthy controls (NP), HD and not-dialysed CKD3-5 patients; fold difference values were calculated by comparison to expression of the target transcript in the reference sample and for the evaluation purposes was set as 1; medians with IRQs; p values *<0.01, **<0.001 and ***<0.0001 indicate statistical significance (Bonferroni correction was applied).
